# Chemical Reactivity Theory Study of Advanced Glycation Endproduct Inhibitors

**DOI:** 10.3390/molecules22020226

**Published:** 2017-02-02

**Authors:** Juan Frau, Daniel Glossman-Mitnik

**Affiliations:** 1 Departament de Química, Universitat de les Illes Balears, 07122 Palma de Mallorca, Spain; juan.frau@uib.es; 2 Laboratorio Virtual NANOCOSMOS, Centro de Investigación en Materiales Avanzados, Departamento de Medio Ambiente y Energía, Chihuahua, Chih 31136, Mexico

**Keywords:** diabetes, Alzheimer, AGEs inhibitors, computational chemistry, molecular modeling, conceptual DFT, chemical reactivity theory

## Abstract

Several compounds with the known ability to perform as inhibitors of advanced glycation endproducts (AGE) have been studied with Density Functional Theory (DFT) through the use of a number of density functionals whose accuracy has been tested across a broad spectrum of databases in Chemistry and Physics. The chemical reactivity descriptors for these systems have been calculated through Conceptual DFT in an attempt to relate their intrinsic chemical reactivity with the ability to inhibit the action of glycating carbonyl compounds on amino acids and proteins. This knowledge could be useful in the design and development of new drugs which can be potential medicines for diabetes and Alzheimer’s disease.

## 1. Introduction

It is well established that advanced glycation endproducts (AGEs) are the final products of a series of chemical reactions initiated by the attachment of reducing sugars to free amino groups in proteins, lipids, and nucleic acids [1,2]. AGEs are a group of complex and heterogeneous compounds that are implicated in diabetic complications and Alzheimer’s disease.

Therefore, there is a large amount of research dedicated to the discovery and investigation of AGE inhibitors that can be used as a therapeutic approach for the treatment of the pathologies associated with them. AGE inhibitors may act by various mechanisms at different steps of advanced glycation endproduct (AGE) formation (depending on oxidative stress and/or carbonyl stress) and AGE-mediated damage: trapping of carbonyl species, antioxidant activity by transition metal chelation, or free radical scavenging.

Chemical Reactivity Theory or Conceptual Density Functional Theory (DFT) [3,4] provides a series of descriptors that can be useful for the analysis of the molecular properties of known AGE inhibitors, and a starting point for the development of new ones. These descriptors are based on the calculation of the energies of the neutral system as well as the corresponding radical cation and anion by means of a ΔSCF technique, that is, the difference in energies between the neutral and the radical ionic species. This can be made simpler by applying the so-called KID procedure (for “Koopmans in DFT”) that consists of the identification of the vertical ionization potential I with $-\epsilon_{H}$ (the energy of the HOMO) and the vertical electron affinity A with $-\epsilon_{L}$ (the energy of the LUMO). This KID procedure is an approximation, because it is well known that the Koopmans’s theorem is not valid within DFT [5,6,7,8]. Notwithstanding, it can be useful for faster calculations of Conceptual DFT descriptors for large molecular systems where the determination of the electronic energy of the radical cation and anion could be computationally costly and difficult to converge.

As a follow-up of our recently published work on the chemistry of antioxidant molecules [9], the objective of this work is to perform a comparative study of the performance of several model chemistries based on the latest Minnesota density functionals [10] for the prediction of the chemical reactivity of twelve inhibitors of advanced glycation endproducts presented in the literature [11,12]. The comparison will be performed through the calculation of the global descriptors that arise from Conceptual DFT and the verification of the KID procedure. It must be stressed that it is not our intention to calculate the vertical I and A to compare them to experimental results, but to predict trends of chemical reactivity that can be useful for other researchers in order to develop new AGE inhibitors.

## 2. Theoretical Background

As this work is part of an ongoing project, the theoretical background is similar to that presented in previous research [9,13,14,15,16,17,18], and will be shown here for the sake of completeness. Within the conceptual framework of DFT [19,20], the chemical potential *μ* is defined as

(1) $$\mu ={\left(\frac{\partial E}{\partial N}\right)}_{v(\vec{r})}=-\chi$$

where *χ* is the electronegativity, while the global hardness *η* is

(2) $$\eta ={\left(\frac{\partial^{2}E}{\partial N^{2}}\right)}_{v(\vec{r})}$$

Using a finite difference approximation and the KID procedure, the above expressions can be written as:

(3) $$\mu =-\frac{1}{2}\left(I+A\right)\approx \frac{1}{2}\left(\epsilon_{L}+\epsilon_{H}\right)=\chi_{K}$$

(4) $$\eta =\left(I-A\right)\approx \left(\epsilon_{L}-\epsilon_{H}\right)=\eta_{K}$$

where $\epsilon_{H}$ and $\epsilon_{L}$ are the energies of the highest occupied and the lowest unoccupied molecular orbitals (HOMO and LUMO), respectively.

The electrophilicity index *ω* has been defined as [21]:

(5) $$\omega =\frac{\mu^{2}}{2\eta }=\frac{{\left(I+A\right)}^{2}}{4(I-A)}\approx \frac{{\left(\epsilon_{L}+\epsilon_{H}\right)}^{2}}{4(\epsilon_{L}-\epsilon_{H})}=\omega_{K}$$

The electrodonating ($\omega^{-}$) and electroaccepting ($\omega^{+}$) powers have been defined as [22]:

(6) $$\omega^{-}=\frac{{\left(3I+A\right)}^{2}}{16(I-A)}\approx \frac{{\left(3\epsilon_{H}+\epsilon_{L}\right)}^{2}}{16\eta_{K}}=\omega_{K}^{-}$$

(7) $$\omega^{+}=\frac{{\left(I+3A\right)}^{2}}{16(I-A)}\approx \frac{{\left(\epsilon_{H}+3\epsilon_{L}\right)}^{2}}{16\eta_{K}}=\omega_{K}^{+}$$

It follows that a larger $\omega^{+}$ value corresponds to a better capability of accepting charge, whereas a smaller value of $\omega^{-}$ makes it a better electron donor. In order to compare $\omega^{+}$ with -$\omega^{-}$, the following definition of net electrophilicity has been proposed [23]:

(8) $$\Delta \omega^{\pm }=\omega^{+}-\left(-\omega^{-}\right)=\omega^{+}+\omega^{-}\approx \omega_{K}^{+}-\left(-\omega_{K}^{-}\right)=\omega_{K}^{+}+\omega_{K}^{-}=\Delta \omega_{K}^{\pm }$$

that is, the electroaccepting power relative to the electrodonating power.

## 3. Settings and Computational Methods

Along the lines of our previous work [9,13,14,15,16,17,18], the computational studies were performed with the Gaussian 09 [24] series of programs, with density functional methods as implemented in the computational package. The equilibrium geometries of the molecules were determined by means of the gradient technique. The force constants and vibrational frequencies were determined by computing analytical frequencies on the stationary points obtained after the optimization to check if there were true minima. The basis set used in this work was Def2SVP for geometry optimization and frequencies, while Def2TZVP was considered for the calculation of the electronic properties [25,26].

For the calculation of the molecular structure and properties of the studied systems [9,13,14,15,16,17,18], we chose several density functionals from the latest Minnesota density functionals family, which consistently provide satisfactory results for several structural and thermodynamic properties [10]: M11, which is a is a range-separated hybrid meta-GGA [27]; M11L, which is a dual-range local meta-GGA [28]; MN12L, which is a nonseparable local meta-NGA [29]; MN12SX, which is a range-separated hybrid nonseparable meta-NGA [30]; N12, which is a nonseparable gradient approximation [31]; N12SX, which is a range-separated hybrid nonseparable gradient approximation [30]; SOGGA11, which is a GGA density functional [32]; and SOGGA11X, which is a hybrid GGA density functional [33]. In these functionals, GGA stands for generalized gradient approximation (in which the density functional depends on the up and down spin densities and their reduced gradient), and NGA stands for nonseparable gradient approximation (in which the density functional depends on the up/down spin densities and their reduced gradient, and also adopts a nonseparable form). All calculations were performed in the presence of water as a solvent, by doing IEF-PCM (Integral Equation Formalism for the Polarized Continuum Model) computations according to the Solvation Model Density (SMD) solvation model [34].

## 4. Results and Discussion

**Figure 1:**
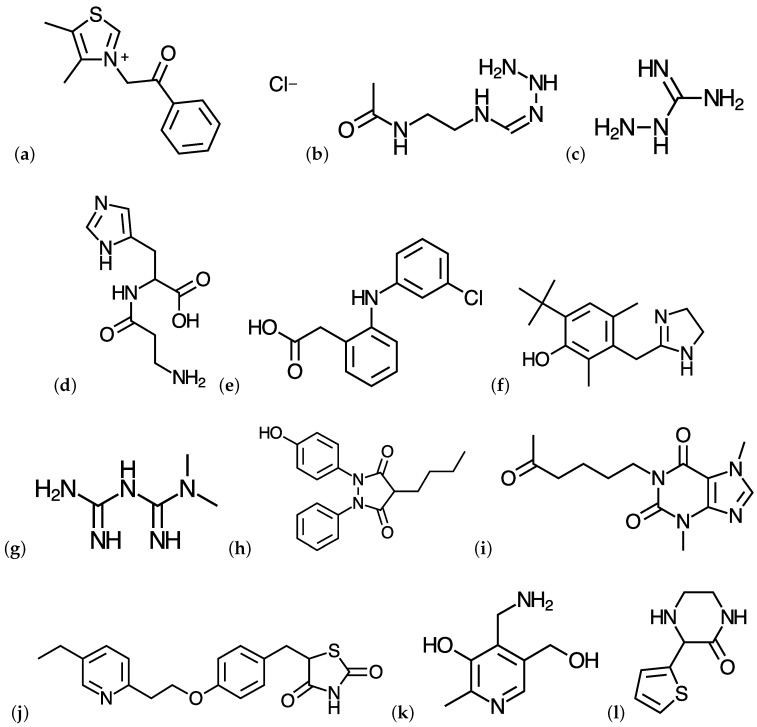
Molecular Structures of (**a**) ALT-711; (**b**) ALT-946; (**c**) Aminoguanidine; (**d**) Carnosine; (**e**) GLY-230; (**f**) LR-9; (**g**) Metformin; (**h**) OPB-9195; (**i**) Pentoxifylline; (**j**) Pioglitazone; (**k**) Pyridoxamine; and (**l**) Tenilsetam.

The molecular structures of ALT-711, ALT-946, aminoguanidine, carnosine, GLY-230, LR-9, metformin, OPB-9195, pentoxifylline, pioglitazone, pyridoxamine, and tenilsetam [11,12] were pre-optimized by starting with the readily available structures shown in Figure 1 and finding the most stable conformers by means of the Avogadro 1.2.0 program [35,36] through a random sampling with molecular mechanics techniques and a consideration of all the torsional angles through the general AMBER force field [37]. The structures of the resulting conformers were then reoptimized with the eight density functionals mentioned in the previous section in conjunction with the Def2SVP basis set and the SMD solvation model, using water as a solvent.

It is worth calculating the electronegativity *χ*, the global hardness *η*, and the global electrophilicity *ω* for the studied systems using the ΔSCF and KID in order to verify the quality of the procedures. Additionally, we will include the electrodonating ($\omega^{-}$) and electroaccepting ($\omega^{+}$) powers as well as the net electrophilicity $\Delta \omega^{\pm }$ in the calculations for further verification.

The HOMO and LUMO orbital energies (in eV), ionization potentials I, electron affinities A (in eV), and global electronegativity *χ*, total hardness *η*, global electrophilicity *ω*, electrodonating power, ($\omega^{-}$), electroaccepting power ($\omega^{+}$), and net electrophilicity $\Delta \omega^{\pm }$ of the AGE inhibitors calculated with the eight density functionals and the Def2TZVP basis set using water as solvent simulated with the SMD parametrization of the IEF-PCM model are presented in Tables S1A to S8A of the Electronic Supplementary Materials (ESM). The upper part of the tables shows the results derived assuming the validity of the KID procedure (hence the subscript K), and the lower part shows the results derived from the calculated ΔSCF energies.

We have previously designed several descriptors that relate the results obtained through the HOMO and LUMO calculations with those obtained by means of the vertical I and A with a ΔSCF procedure. However, it must be stressed that it is not our intention to perform a gap-fitting by minimizing a descriptor by choosing optimal range-separation parameter *γ*, but to check if the density functionals considered in this study—in which, some of them contain a fixed range-separation parameter *γ*—obey the KID procedure. In fact, there is no range-separation parameter *γ* in our designed descriptors. Moreover, we have considered A as minus the energy of the LUMO of the neutral system instead of considering A as minus the energy of the HOMO of the N+1 electron system, as it was in References [38,39].

The first three descriptors are related to the simplest fulfillment of the KID procedure by relating $\epsilon_{H}$ with -I, $\epsilon_{L}$ with -A, and the behavior of them in the description of the HOMO–LUMO gap: $J_{I}=\left|\epsilon_{H}+E_{gs}\left(N-1\right)-E_{gs}\left(N\right)\right|$, $J_{A}=\left|\epsilon_{L}+E_{gs}\left(N\right)-E_{gs}\left(N+1\right)\right|$, and $J_{HL}=\sqrt{{J_{I}}^{2}+{J_{A}}^{2}}$.

Next, we consider four other descriptors that analyze how well the studied density functionals are useful for the prediction of the electronegativity *χ*, the global hardness *η*, and the global electrophilicity *ω*, and for a combination of these Conceptual DFT descriptors, just considering the energies of the HOMO and LUMO or the vertical I and A: $J_{\chi }=\left|\chi -\chi_{K}\right|$, $J_{\eta }=\left|\eta -\eta_{K}\right|$, $J_{\omega }=\left|\omega -\omega_{K}\right|$ and $J_{D1}=\sqrt{J_{\chi }^{2}+J_{\eta }^{2}+J_{\omega }^{2}}$, where D1 stands for the first group of Conceptual DFT descriptors.

Finally, we designed four other descriptors to verify the goodness of the studied density functionals for the prediction of the electroaccepting power $\omega^{+}$, the electrodonating power $\omega^{-}$, the net electrophilicity $\Delta \omega^{\pm }$ , and for a combination of these Conceptual DFT descriptors, just considering the energies of the HOMO and LUMO or the vertical I and A: $J_{\omega^{+}}=\left|\omega^{+}-\omega_{K}^{+}\right|$, $J_{\omega^{-}}=\left|\omega^{-}-\omega_{K}^{-}\right|$, $J_{\Delta \omega^{\pm }}=\left|\Delta \omega^{\pm }-\Delta \omega_{K}^{\pm }\right|$ and $J_{D2}=\sqrt{J_{\omega^{-}}^{2}+J_{\omega^{+}}^{2}+J_{\Delta \omega^{\pm }}^{2}}$, where D2 stands for the first group of Conceptual DFT descriptors.

The results of the calculations of J${}_{I}$, J${}_{A}$, J${}_{HL}$, J${}_{\chi }$, J${}_{\eta }$, J${}_{\omega }$, J${}_{D1}$, J${}_{\omega^{+}}$, J${}_{\omega^{-}}$, J${}_{\Delta \omega^{\pm }}$, and J${}_{D2}$ for the AGE inhibitors considered in this work are displayed in Tables S1B to S8B of the Electronic Supplementary Materials.

On the basis of the results for the descriptors presented on Tables S1B–S8B of the ESM, we have compiled the average values for for each density functional on the whole group of AGE inhibitors, and the calculated results are displayed on Table 1.

As can be seen from Table 1, the KID procedure holds with great accuracy for the MN12SX and N12SX density functionals, which are a range-separated hybrid meta-NGA and a range-separated hybrid NGA density functionals, respectively. Indeed, the values of J${}_{I}$, J${}_{A}$, and J${}_{HL}$ are not exactly zero. However, their values can be favorably compared with the results presented for these quantities in the work of Lima et al. [39], where the minima have been obtained by choosing a parameter that enforces that behavior.

It is interesting to see that the same density functionals also fulfill the KID procedure for the other descriptors, namely J${}_{\chi }$, J${}_{\eta }$, J${}_{\omega }$, and J${}_{D1}$, as well as for J${}_{\omega^{-}}$, J${}_{\omega^{+}}$, J${}_{\Delta \omega^{\pm }}$, and J${}_{D2}$. These results are very important, because they show that it is not enough to rely only on J${}_{I}$, J${}_{A}$, and J${}_{HL}$. For example, if we consider only J${}_{\chi }$, the values are very close to zero for all of the density functionals considered. As for the other descriptors, only the MN12SX and N12SX density functionals show this behavior. This means that the results for J${}_{\chi }$ are due to a fortuitous cancellation of errors.

The usual GGA (SOGGA11) and hybrid-GGA (SOGGA11X) are not good for the fulfillment of the KID procedure, and the same conclusion is valid for the local functionals M11L, MN12L and N12.

An important fact is that although the range-separated hybrid NGA and range-separated hybrid meta-NGA density functionals can be useful for the calculation of the Conceptual DFT descriptors, it is not the same for the range-separated hybrid GG A (M11) density functional. An inspection of Table S1A of the ESM shows that this is due to the fact that this functional inadequately describes the energy of the LUMO, leading to negative values of A, which are in contradiction with the ΔSCF results.

As the mode of action of the studied AGE inhibitors can be different, involving one, two or three of the mechanisms presented in the Introduction, it is very difficult to say which of them will be more effective as an inhibitor of the glycation process. However, in a recent experimental work on the reactivity, selectivity, and reaction mechanisms of some sequestering agents of reactive carbonyl species [40], the authors attempted to relate their results with several Conceptual DFT descriptors. Although only three of our studied AGEs inhibitors were considered in that work, it is worth seeing if a similar comparison can be performed using the results of our calculations. Notwithstanding, it is not fair to attempt a linear regression analysis with only three points, but potentially good qualitative trends for their ability to perform as inhibitors of AGEs could be probably obtained.

Indeed, the key factors in the study of the reactivity of the considered AGEs inhibitors is their nucleophilic character, because we are only assuming the interaction through the Maillard reaction. Thus, we are going to consider the published results for the interaction of the sequestering agents with methylglyoxal (MGO). There are several definitions of nucleophilicity available in the literature of Conceptual DFT, and the interested reader is referred to the recent work of Domingo and Pérez [41]. For our purposes, the most significant definitions are those that relate the nucleophilicity N with the inverse of the electrophilicity *ω* [42] or with the inverse of the electrodonating power $\omega^{-}$ [43]. It is worth observing that the trend for the ability to react with glycating carbonyls that arises from Table 1 of the work of Colzani et al. [40]—that is, aminoguanidine > carnosine > pyridoxamine—correlates well with the previous definitions of nucleophilicity N, as calculated from the results of Tables S4A and S6A of the ESM. It is interesting to see that the same correlations are found if we consider the inverse of the net electrophilicity $\Delta \omega^{\pm }$ results that are shown in Tables S4A and S6A of the ESM. This suggests that the inverse of $\Delta \omega^{\pm }$ could be an alternative definition for the nucleophilicity N.

On the basis of the previous analysis, and extending the correlation to the other molecular systems under study, we can find the approximate qualitative trends:

ALT−946>Aminoguanidine>Metformin>Carnosine>GLY−230>Tenilsetam>

>Pyridoxamine>Pentoxifylline≈Pioglitazone>LR−9>OPB−9195>ALT−711

for the MN12SX density functional, and

Aminoguanidine>ALT−946>Metformin>Carnosine>GLY−230>Tenilsetam>

>Pyridoxamine>Pentoxifylline≈Pioglitazone>LR−9>OPB−9195>ALT−711

for the N12SX density functional.

These qualitative trends are very similar (except for the different rank of aminoguanidine in each case), and they are representative of the known pharmacological properties of the studied AGE inhibitors [11,12]. Therefore, the calculation of the nucleophilicity N (as the inverse of *ω*, the inverse of $\omega^{-}$, or probably the inverse of $\Delta \omega^{\pm }$) within both model chemistries could be useful for the prediction of the AGE inhibition ability of potential molecular systems designed with this purpose in mind.

## 5. Conclusions

The latest Minnesota family of density functionals (M11, M11L, MN12L, MN12SX, N12, N12SX, SOGGA11, and SOGGA11X) have been tested for the fulfillment of the KID procedure by comparison of the HOMO- and LUMO-derived values with those obtained through a ΔSCF technique. It has been shown that the range-separated hybrid meta-NGA density functional (MN12SX) and the range-separated hybrid NGA density functional (N12SX) are the best for the accomplishment of this objective. As such, they are a good alternative to those density functionals whose behavior have been tuned through a gap-fitting procedure and a good prospect for their usefulness in the description of the chemical reactivity of molecular systems of large size.

From the whole of the results presented in this work, it can be seen that the sites of interaction of the studied AGE inhibitors can be predicted by using DFT-based reactivity descriptors such as the electronegativity, global hardness, global electrophilicity, electrodonating and electroaccepting powers, and net electrophilicity. These descriptors were used in the characterization and successful description of the proposed AGE inhibitors, and provide a firm explanation for the reactivity of those molecules.

Moreover, a qualitative trend has been found between the potential pharmacological activity and the nucleophilicity N of the studied molecules. This is based on calculations performed with the MN12SX and N12SX density functionals in connection with the Def2TZVP basis set and the SMD parametrization of the IEF-PCM model using water as a solvent. It can be concluded that these model chemistries (MN12SX/Def2TZVP/SMD(Water) and N12SX/Def2TZVP/SMD(Water)) are the best for fulfilling the KID procedure and for the prediction of the of the potential AGE inhibition ability of molecular systems.

## Figures and Tables

**Table 1:** Average descriptors J${}_{I}$, J${}_{A}$, J${}_{HL}$, J${}_{\chi }$, J${}_{\eta }$, J${}_{\omega }$, J${}_{D1}$, J${}_{\omega^{+}}$, J${}_{\omega^{-}}$, J${}_{\Delta \omega^{\pm }}$, and J${}_{D2}$ for the ALT-711, ALT-946, aminoguanidine, carnosine, GLY-230, LR-9, metformin, OPB-9195, pentoxifylline, pioglitazone, pyridoxamine, and tenilsetam calculated with the M11, M11L, MN12L, MN12SX, N12, N12SX, SOGGA11, and SOGGA11X density functionals and the Def2TZVP basis set using water as solvent simulated with the solvation model density (SMD) parametrization of the IEF-PCM (Integral Equation Formalism for the Polarized Continuum Model) model.

	J${}_{I}$	J${}_{A}$	J${}_{\mathrm{HL}}$	J${}_{\chi }$	J${}_{\eta }$	J${}_{\omega }$	J${}_{D1}$	J${}_{\omega^{-}}$	J${}_{\omega^{+}}$	J${}_{\Delta \omega^{\pm }}$	J${}_{D2}$
M11	2.446	2.333	3.382	0.074	4.779	0.661	4.838	0.999	1.052	2.046	2.513
M11L	0.352	0.196	0.420	0.092	0.548	0.181	0.601	0.344	0.347	0.681	0.846
MN12L	0.311	0.198	0.377	0.072	0.491	0.133	0.528	0.242	0.249	0.487	0.604
MN12SX	0.069	0.108	0.143	0.067	0.125	0.054	0.164	0.137	0.070	0.207	0.260
N12	0.459	0.355	0.601	0.088	0.811	0.306	0.896	0.568	0.567	1.135	1.396
N12SX	0.070	0.102	0.146	0.054	0.164	0.043	0.184	0.103	0.068	0.156	0.204
SOGGA11	0.489	0.448	0.699	0.134	0.937	0.412	1.060	0.809	0.759	1.551	1.918
SOGGA11X	0.944	0.958	1.346	0.032	1.902	0.381	1.953	0.646	0.639	1.286	1.575

## Supplementary Materials

The following are available online at: http://www.mdpi.com/1420-3049/22/2/226/s1, ESM.pdf : Electronic Supplementary Materials.

## Abbreviations

The following abbreviations are used in this manuscript:

DFT	Density Functional Theory
AGE	Advanced Glycation Endproducts
SCF	Self Consistent Field
KID	Koopmans in DFT
HOMO	Higher Ocuppied Molecular Orbital
LUMO	Lower Unocuppied Molecular Orbital
IEF-PCM	Integral Equation Formalism - Polarized Continuum Model
SMD	Solvation Model Density
