# Supplementary material for: Chemical Reactivity Theory Study of Advanced Glycation Endproduct Inhibitors
Source: Molecules. 2017 Feb 2;22(2):226. doi: 10.3390/molecules22020226 (PMC6155735; doi:10.3390/molecules22020226)
Supplement: Supplementary file 1 [file molecules-22-00226-s001.pdf]

Chemical Reactivity Theory Study of Advanced Glycation Endproducts Inhibitors

Electronic Supplementary Materials

Juan Frau and Daniel Glossman-Mitnik

Table S1A: HOMO and LUMO orbital energies (in eV), ionization potentials I and electron affinities A (in eV), and global electronegativity χ, total hardness η, global electrophilicity ω, electrodonating power (ω^-), electroaccepting power (ω^+), and net electrophilicity $\Delta\omega^\pm$ of ALT-711, ALT-946, Aminoguanidine, Carnosine, GLY-230, LR-9, Metformin, OPB-9195, Pentoxifylline, Pioglitazone, Pyridoxamine and Tenilsetam calculated with the M11 density functional and the Def2TZVP basis set using water as solvent simulated with the SMD parametrization of the IEF-PCM model. The upper part of the table shows the results derived assuming the validity of the KID procedure and the lower part shows the results derived from the calculated ΔSCF energies.

Property	HOMO	LUMO	χ_K	η_K	ω_K	ω_K^-	ω_K^+	$\Delta\omega_K^\pm$
ALT-711	-8.677	0.029	4.324	8.706	1.074	4.854	0.530	5.384
ALT-946	-8.023	2.538	2.743	10.561	0.356	2.744	0.001	2.745
Aminoguanidine	-8.174	2.736	2.719	10.910	0.339	2.719	0.000	2.719
Carnosine	-8.794	1.911	3.442	10.705	0.553	3.497	0.055	3.551
GLY-230	-8.100	1.219	3.440	9.319	0.635	3.573	0.132	3.705
LR-9	-8.323	0.149	4.087	8.472	0.986	4.544	0.458	5.002
Metformin	-8.692	2.374	3.159	11.066	0.451	3.173	0.014	3.187
OPB-9195	-8.822	0.124	4.349	8.946	1.057	4.848	0.499	5.347
Pentoxifylline	-8.726	0.920	3.903	9.646	0.789	4.133	0.231	4.364
Pioglitazone	-8.690	0.947	3.871	9.637	0.778	4.093	0.222	4.315
Pyridoxamine	-8.818	1.224	3.797	10.042	0.718	3.962	0.165	4.127
Tenilsetam	-9.018	1.434	3.792	10.452	0.688	3.925	0.133	4.059
Property	I	A	χ	η	ω	ω^-	ω^+	$\Delta\omega^\pm$
ALT-711	6.417	2.266	4.342	4.151	2.271	6.971	2.630	9.601
ALT-946	5.505	-0.443	2.531	5.948	0.539	2.714	0.183	2.898
Aminoguanidine	5.515	-0.384	2.565	5.899	0.558	2.767	0.202	2.968
Carnosine	6.201	0.793	3.497	5.408	1.131	4.348	0.851	5.199
GLY-230	5.818	0.785	3.302	5.033	1.083	4.131	0.830	4.961
LR-9	6.056	2.002	4.029	4.054	2.002	6.272	2.243	8.515
Metformin	6.051	0.062	3.057	5.988	0.780	3.463	0.406	3.869
OPB-9195	6.454	2.246	4.350	4.207	2.249	6.936	2.586	9.521
Pentoxifylline	6.389	1.444	3.917	4.945	1.551	5.370	1.453	6.823
Pioglitazone	6.264	1.509	3.886	4.755	1.588	5.417	1.531	6.948
Pyridoxamine	6.336	1.153	3.744	5.183	1.352	4.901	1.157	6.057
Tenilsetam	6.495	0.953	3.724	5.542	1.251	4.711	0.987	5.697

Table S1B: Descriptors J_I, J_A, J_{HL}, J_χ, J_η, J_ω, J_{D1}, J_{ω^+}, J_{ω^-}, $J_{\Delta\omega^\pm}$ and J_{D2} for the ALT-711, ALT-946, Aminoguanidine, Carnosine, GLY-230, LR-9, Metformin, OPB-9195, Pentoxifylline, Pioglitazone, Pyridoxamine and Tenilsetam molecules calculated from the results of Table S1A

	J_I	J_A	J_{HL}	J_χ	J_η	J_ω	J_{D1}	J_{ω^-}	J_{ω^+}	$J_{\Delta\omega^\pm}$	J_{D2}
ALT-711	2.260	2.295	3.221	0.018	4.555	1.197	4.709	2.118	2.100	4.217	5.165
ALT-946	2.518	2.095	3.275	0.212	4.613	0.182	4.621	0.029	0.182	0.153	0.240
Aminoguanidine	2.660	2.352	3.550	0.154	5.012	0.219	5.019	0.048	0.202	0.249	0.324
Carnosine	2.593	2.704	3.746	0.055	5.297	0.577	5.328	0.851	0.796	1.647	2.018
GLY-230	2.282	2.004	3.037	0.139	4.286	0.448	4.312	0.558	0.697	1.255	1.541
LR-9	2.267	2.151	3.125	0.058	4.418	1.016	4.534	1.728	1.786	3.513	4.303
Metformin	2.641	2.436	3.593	0.102	5.077	0.329	5.089	0.290	0.392	0.682	0.839
OPB-9195	2.369	2.371	3.351	0.001	4.739	1.192	4.887	2.088	2.087	4.175	5.113
Pentoxifylline	2.337	2.365	3.324	0.014	4.701	0.762	4.763	1.237	1.223	2.460	3.012
Pioglitazone	2.426	2.456	3.452	0.015	4.882	0.811	4.949	1.324	1.309	2.633	3.224
Pyridoxamine	2.483	2.376	3.437	0.053	4.859	0.634	4.900	0.939	0.992	1.930	2.364
Tenilsetam	2.523	2.387	3.473	0.068	4.910	0.563	4.943	0.785	0.854	1.639	2.008
Average	2.446	2.333	3.382	0.074	4.779	0.661	4.838	0.999	1.052	2.046	2.513

Table S2A: HOMO and LUMO orbital energies (in eV), ionization potentials I and electron affinities A (in eV), and global electronegativity χ, total hardness η, global electrophilicity ω, electrodonating power (ω^-), electroaccepting power (ω^+), and net electrophilicity $\Delta\omega^\pm$ of ALT-711, ALT-946, Aminoguanidine, Carnosine, GLY-230, LR-9, Metformin, OPB-9195, Pentoxifylline, Pioglitazone, Pyridoxamine and Tenilsetam calculated with the M11L density functional and the Def2TZVP basis set using water as solvent simulated with the SMD parametrization of the IEF-PCM model. The upper part of the table shows the results derived assuming the validity of the KID procedure and the lower part shows the results derived from the calculated ΔSCF energies.

Property	HOMO	LUMO	χ_K	η_K	ω_K	ω_K^-	ω_K^+	$\Delta\omega_K^\pm$
ALT-711	-5.878	-2.651	4.264	3.227	2.817	7.968	3.704	11.673
ALT-946	-5.090	-0.378	2.734	4.712	0.793	3.247	0.514	3.761
Aminoguanidine	-4.967	-0.042	2.505	4.925	0.637	2.834	0.329	3.163
Carnosine	-5.650	-0.981	3.316	4.669	1.177	4.304	0.988	5.292
GLY-230	-5.218	-1.496	3.357	3.721	1.514	4.940	1.582	6.522
LR-9	-5.450	-2.459	3.955	2.991	2.614	7.393	3.438	10.831
Metformin	-5.536	-0.380	2.958	5.156	0.849	3.499	0.540	4.039
OPB-9195	-5.733	-2.661	4.197	3.072	2.867	8.025	3.827	11.852
Pentoxifylline	-5.760	-1.699	3.729	4.061	1.713	5.544	1.814	7.358
Pioglitazone	-5.791	-1.724	3.758	4.068	1.736	5.604	1.847	7.451
Pyridoxamine	-5.759	-1.551	3.655	4.208	1.587	5.265	1.610	6.875
Tenilsetam	-5.891	-1.403	3.647	4.488	1.482	5.068	1.421	6.489
Property	I	A	χ	η	ω	ω^-	ω^+	$\Delta\omega^\pm$
ALT-711	6.632	2.442	4.537	4.190	2.456	7.443	2.906	10.349
ALT-946	5.502	0.253	2.877	5.250	0.789	3.344	0.467	3.811
Aminoguanidine	5.438	0.038	2.738	5.400	0.694	3.095	0.357	3.452
Carnosine	5.931	0.830	3.381	5.101	1.120	4.249	0.869	5.118
GLY-230	5.473	1.335	3.404	4.137	1.400	4.761	1.357	6.119
LR-9	5.699	2.241	3.970	3.458	2.279	6.760	2.789	9.549
Metformin	5.888	0.309	3.099	5.578	0.861	3.619	0.521	4.140
OPB-9195	6.024	2.326	4.175	3.698	2.357	7.033	2.858	9.891
Pentoxifylline	6.100	1.434	3.767	4.666	1.521	5.217	1.450	6.667
Pioglitazone	6.070	1.578	3.824	4.491	1.628	5.449	1.625	7.073
Pyridoxamine	6.005	1.251	3.628	4.754	1.384	4.879	1.252	6.131
Tenilsetam	6.192	1.040	3.616	5.152	1.269	4.668	1.052	5.720

Table S2B: Descriptors J_I, J_A, J_{HL}, J_χ, J_η, J_ω, J_{D1}, J_{ω^+}, J_{ω^-}, $J_{\Delta\omega^\pm}$ and J_{D2} for the ALT-711, ALT-946, Aminoguanidine, Carnosine, GLY-230, LR-9, Metformin, OPB-9195, Pentoxifylline, Pioglitazone, Pyridoxamine and Tenilsetam molecules calculated from the results of Table S2A

	J_I	J_A	J_{HL}	J_χ	J_η	J_ω	J_{D1}	J_{ω^-}	J_{ω^+}	$J_{\Delta\omega^\pm}$	J_{D2}
ALT-711	0.754	0.209	0.783	0.273	0.963	0.361	1.064	0.526	0.798	1.324	1.633
ALT-946	0.413	0.125	0.431	0.144	0.538	0.004	0.557	0.097	0.047	0.050	0.119
Aminoguanidine	0.471	0.004	0.471	0.234	0.475	0.057	0.533	0.261	0.028	0.289	0.390
Carnosine	0.281	0.151	0.319	0.065	0.432	0.057	0.440	0.055	0.119	0.174	0.218
GLY-230	0.255	0.161	0.301	0.047	0.416	0.114	0.434	0.178	0.225	0.403	0.495
LR-9	0.249	0.218	0.331	0.015	0.467	0.335	0.575	0.633	0.649	1.282	1.570
Metformin	0.351	0.071	0.359	0.140	0.422	0.012	0.445	0.121	0.020	0.101	0.158
OPB-9195	0.291	0.335	0.444	0.022	0.626	0.510	0.807	0.992	0.970	1.961	2.402
Pentoxifylline	0.341	0.265	0.432	0.038	0.606	0.192	0.636	0.327	0.365	0.691	0.847
Pioglitazone	0.278	0.145	0.314	0.066	0.424	0.108	0.442	0.156	0.222	0.378	0.465
Pyridoxamine	0.246	0.300	0.388	0.027	0.546	0.203	0.583	0.385	0.358	0.744	0.911
Tenilsetam	0.300	0.363	0.471	0.031	0.664	0.213	0.698	0.400	0.369	0.769	0.943
Average	0.352	0.196	0.420	0.092	0.548	0.181	0.601	0.344	0.347	0.681	0.846

Table S3A: HOMO and LUMO orbital energies (in eV), ionization potentials I and electron affinities A (in eV), and global electronegativity χ, total hardness η, global electrophilicity ω, electrodonating power (ω^-), electroaccepting power (ω^+), and net electrophilicity $\Delta\omega^\pm$ of ALT-711, ALT-946, Aminoguanidine, Carnosine, GLY-230, LR-9, Metformin, OPB-9195, Pentoxifylline, Pioglitazone, Pyridoxamine and Tenilsetam calculated with the MN12L density functional and the Def2TZVP basis set using water as solvent simulated with the SMD parametrization of the IEF-PCM model. The upper part of the table shows the results derived assuming the validity of the KID procedure and the lower part shows the results derived from the calculated ΔSCF energies.

Property	HOMO	LUMO	χ_K	η_K	ω_K	ω_K^-	ω_K^+	$\Delta\omega_K^\pm$
ALT-711	-5.417	-2.424	3.921	2.993	2.568	7.283	3.362	10.645
ALT-946	-4.738	0.071	2.333	4.809	0.566	2.599	0.266	2.865
Aminoguanidine	-4.711	0.553	2.079	5.265	0.411	2.190	0.111	2.300
Carnosine	-5.467	-0.622	3.045	4.845	0.957	3.739	0.694	4.433
GLY-230	-5.031	-1.005	3.018	4.026	1.131	4.023	1.005	5.028
LR-9	-5.252	-2.043	3.647	3.209	2.072	6.169	2.522	8.691
Metformin	-5.320	0.136	2.592	5.456	0.616	2.868	0.276	3.145
OPB-9195	-5.689	-2.276	3.982	3.413	2.324	6.851	2.869	9.721
Pentoxifylline	-5.577	-1.324	3.451	4.253	1.400	4.790	1.340	6.130
Pioglitazone	-5.548	-1.321	3.435	4.227	1.395	4.772	1.338	6.110
Pyridoxamine	-5.607	-1.100	3.353	4.508	1.247	4.453	1.100	5.553
Tenilsetam	-5.742	-0.963	3.352	4.779	1.176	4.327	0.974	5.301
Property	I	A	χ	η	ω	ω^-	ω^+	$\Delta\omega^\pm$
ALT-711	6.030	2.204	4.117	3.826	2.215	6.728	2.611	9.339
ALT-946	5.115	-0.209	2.453	5.324	0.565	2.689	0.236	2.925
Aminoguanidine	5.125	-0.448	2.338	5.573	0.491	2.499	0.160	2.659
Carnosine	5.781	0.449	3.115	5.331	0.910	3.711	0.596	4.307
GLY-230	5.250	0.885	3.067	4.365	1.078	3.962	0.894	4.856
LR-9	5.474	1.864	3.669	3.610	1.864	5.789	2.120	7.909
Metformin	5.561	-0.340	2.610	5.901	0.577	2.829	0.218	3.047
OPB-9195	5.894	1.992	3.943	3.903	1.992	6.199	2.256	8.455
Pentoxifylline	5.880	1.077	3.478	4.803	1.259	4.558	1.080	5.638
Pioglitazone	5.816	1.165	3.490	4.651	1.309	4.655	1.165	5.819
Pyridoxamine	5.876	0.836	3.356	5.041	1.117	4.227	0.871	5.098
Tenilsetam	6.027	0.676	3.352	5.351	1.050	4.110	0.758	4.868

Table S3B: Descriptors J_I, J_A, J_{HL}, J_χ, J_η, J_ω, J_{D1}, J_{ω^+}, J_{ω^-}, $J_{\Delta\omega^\pm}$ and J_{D2} for the ALT-711, ALT-946, Aminoguanidine, Carnosine, GLY-230, LR-9, Metformin, OPB-9195, Pentoxifylline, Pioglitazone, Pyridoxamine and Tenilsetam molecules calculated from the results of Table S3A

	J_I	J_A	J_{HL}	J_χ	J_η	J_ω	J_{D1}	J_{ω^-}	J_{ω^+}	$J_{\Delta\omega^\pm}$	J_{D2}
ALT-711	0.613	0.220	0.651	0.196	0.832	0.352	0.925	0.555	0.751	1.306	1.605
ALT-946	0.377	0.138	0.401	0.119	0.515	0.001	0.528	0.090	0.030	0.060	0.112
Aminoguanidine	0.413	0.105	0.427	0.259	0.308	0.080	0.411	0.309	0.050	0.359	0.476
Carnosine	0.314	0.173	0.358	0.070	0.486	0.047	0.494	0.028	0.098	0.126	0.162
GLY-230	0.219	0.120	0.250	0.049	0.339	0.054	0.347	0.062	0.111	0.172	0.214
LR-9	0.222	0.179	0.285	0.022	0.401	0.208	0.452	0.380	0.402	0.782	0.958
Metformin	0.241	0.204	0.316	0.018	0.445	0.038	0.447	0.040	0.058	0.098	0.120
OPB-9195	0.206	0.284	0.351	0.039	0.490	0.332	0.593	0.652	0.613	1.265	1.550
Pentoxifylline	0.303	0.247	0.391	0.028	0.550	0.140	0.568	0.232	0.260	0.493	0.604
Pioglitazone	0.268	0.157	0.310	0.056	0.424	0.086	0.436	0.118	0.173	0.291	0.358
Pyridoxamine	0.269	0.264	0.377	0.002	0.533	0.130	0.549	0.226	0.228	0.455	0.557
Tenilsetam	0.285	0.287	0.405	0.001	0.572	0.126	0.586	0.217	0.216	0.433	0.530
Average	0.311	0.198	0.377	0.072	0.491	0.133	0.528	0.242	0.249	0.487	0.604

Table S4A: HOMO and LUMO orbital energies (in eV), ionization potentials I and electron affinities A (in eV), and global electronegativity χ, total hardness η, global electrophilicity ω, electrodonating power (ω^-), electroaccepting power (ω^+), and net electrophilicity $\Delta\omega^\pm$ of ALT-711, ALT-946, Aminoguanidine, Carnosine, GLY-230, LR-9, Metformin, OPB-9195, Pentoxifylline, Pioglitazone, Pyridoxamine and Tenilsetam calculated with the MN12SX density functional and the Def2TZVP basis set using water as solvent simulated with the SMD parametrization of the IEF-PCM model. The upper part of the table shows the results derived assuming the validity of the KID procedure and the lower part shows the results derived from the calculated ΔSCF energies.

Property	HOMO	LUMO	χ_K	η_K	ω_K	ω_K^-	ω_K^+	$\Delta\omega_K^\pm$
ALT-711	-5.916	-2.263	4.089	3.653	2.289	6.850	2.761	9.611
ALT-946	-5.388	-0.047	2.717	5.341	0.691	3.075	0.358	3.433
Aminoguanidine	-5.429	-0.050	2.740	5.379	0.698	3.101	0.362	3.463
Carnosine	-6.060	-0.561	3.310	5.499	0.996	3.991	0.681	4.673
GLY-230	-5.566	-0.954	3.260	4.611	1.152	4.223	0.963	5.186
LR-9	-5.817	-1.957	3.887	3.859	1.957	6.100	2.213	8.312
Metformin	-5.952	-0.071	3.012	5.881	0.771	3.416	0.404	3.820
OPB-9195	-6.225	-2.168	4.197	4.057	2.171	6.693	2.496	9.190
Pentoxifylline	-6.151	-1.253	3.702	4.899	1.399	4.955	1.253	6.208
Pioglitazone	-6.160	-1.253	3.707	4.907	1.400	4.960	1.254	6.214
Pyridoxamine	-6.197	-1.038	3.618	5.159	1.268	4.668	1.050	5.718
Tenilsetam	-6.317	-0.850	3.584	5.467	1.175	4.483	0.899	5.382
Property	I	A	χ	η	ω	ω^-	ω^+	$\Delta\omega^\pm$
ALT-711	6.227	2.314	4.270	3.913	2.330	7.040	2.769	9.809
ALT-946	5.343	-0.071	2.636	5.414	0.642	2.940	0.304	3.244
Aminoguanidine	5.364	-0.497	2.433	5.861	0.505	2.593	0.160	2.753
Carnosine	6.037	0.621	3.329	5.416	1.023	4.049	0.720	4.768
GLY-230	5.503	1.018	3.260	4.485	1.185	4.280	1.020	5.300
LR-9	5.794	2.016	3.905	3.778	2.018	6.224	2.319	8.543
Metformin	5.803	-0.084	2.859	5.886	0.694	3.187	0.327	3.514
OPB-9195	6.193	2.231	4.212	3.962	2.239	6.832	2.620	9.452
Pentoxifylline	6.143	1.306	3.724	4.837	1.434	5.032	1.307	6.339
Pioglitazone	6.110	1.322	3.716	4.788	1.442	5.042	1.325	6.367
Pyridoxamine	6.149	1.078	3.614	5.071	1.288	4.699	1.086	5.785
Tenilsetam	6.304	0.865	3.584	5.439	1.181	4.494	0.910	5.404

Table S4B: Descriptors J_I, J_A, J_{HL}, J_χ, J_η, J_ω, J_{D1}, J_{ω^+}, J_{ω^-}, $J_{\Delta\omega^\pm}$ and J_{D2} for the ALT-711, ALT-946, Aminoguanidine, Carnosine, GLY-230, LR-9, Metformin, OPB-9195, Pentoxifylline, Pioglitazone, Pyridoxamine and Tenilsetam molecules calculated from the results of Table S4A

	J_I	J_A	J_{HL}	J_χ	J_η	J_ω	J_{D1}	J_{ω^-}	J_{ω^+}	$J_{\Delta\omega^\pm}$	J_{D2}
ALT-711	0.312	0.051	0.316	0.181	0.261	0.041	0.320	0.190	0.008	0.198	0.274
ALT-946	0.045	0.118	0.126	0.081	0.073	0.049	0.120	0.135	0.054	0.189	0.238
Aminoguanidine	0.065	0.547	0.551	0.306	0.482	0.193	0.603	0.508	0.202	0.710	0.896
Carnosine	0.023	0.060	0.064	0.018	0.083	0.027	0.089	0.057	0.039	0.096	0.118
GLY-230	0.063	0.063	0.089	0.000	0.126	0.033	0.130	0.057	0.057	0.115	0.140
LR-9	0.023	0.058	0.063	0.018	0.081	0.060	0.103	0.124	0.107	0.231	0.284
Metformin	0.149	0.155	0.215	0.152	0.006	0.077	0.171	0.229	0.077	0.306	0.390
OPB-9195	0.032	0.063	0.071	0.016	0.095	0.068	0.118	0.139	0.123	0.262	0.321
Pentoxifylline	0.009	0.053	0.053	0.022	0.061	0.035	0.074	0.076	0.054	0.131	0.161
Pioglitazone	0.050	0.069	0.085	0.009	0.119	0.042	0.127	0.081	0.072	0.153	0.188
Pyridoxamine	0.048	0.040	0.063	0.004	0.088	0.019	0.090	0.031	0.035	0.067	0.082
Tenilsetam	0.013	0.015	0.020	0.001	0.028	0.007	0.029	0.012	0.011	0.023	0.028
Average	0.069	0.108	0.143	0.067	0.125	0.054	0.164	0.137	0.070	0.207	0.260

Table S5A: HOMO and LUMO orbital energies (in eV), ionization potentials I and electron affinities A (in eV), and global electronegativity χ, total hardness η, global electrophilicity ω, electrodonating power (ω^-), electroaccepting power (ω^+), and net electrophilicity $\Delta\omega^{\pm}$ of ALT-711, ALT-946, Aminoguanidine, Carnosine, GLY-230, LR-9, Metformin, OPB-9195, Pentoxifylline, Pioglitazone, Pyridoxamine and Tenilsetam calculated with the N12 density functional and the Def2TZVP basis set using water as solvent simulated with the SMD parametrization of the IEF-PCM model. The upper part of the table shows the results derived assuming the validity of the KID procedure and the lower part shows the results derived from the calculated ΔSCF energies.

Property	HOMO	LUMO	χ_K	η_K	ω_K	ω_K^-	ω_K^+	$\Delta\omega_K^{\pm}$
ALT-711	-4.989	-2.674	3.831	2.315	3.170	8.401	4.570	12.971
ALT-946	-4.139	-0.312	2.226	3.828	0.647	2.646	0.421	3.067
Aminoguanidine	-4.281	0.562	1.860	4.844	0.357	1.946	0.087	2.033
Carnosine	-5.194	-1.068	3.131	4.126	1.188	4.200	1.069	5.269
GLY-230	-4.639	-1.223	2.931	3.416	1.258	4.195	1.263	5.458
LR-9	-4.930	-2.198	3.564	2.732	2.325	6.603	3.039	9.643
Metformin	-4.808	-0.220	2.514	4.588	0.689	2.922	0.408	3.329
OPB-9195	-5.287	-2.490	3.888	2.797	2.703	7.525	3.636	11.161
Pentoxifylline	-5.302	-1.676	3.489	3.626	1.678	5.328	1.839	7.167
Pioglitazone	-5.193	-1.884	3.538	3.309	1.892	5.760	2.221	7.981
Pyridoxamine	-5.402	-1.418	3.410	3.984	1.459	4.873	1.463	6.336
Tenilsetam	-5.231	-1.143	3.187	4.088	1.242	4.334	1.147	5.481
Property	I	A	χ	η	ω	ω^-	ω^+	$\Delta\omega^{\pm}$
ALT-711	5.953	2.289	4.121	3.663	2.318	6.925	2.804	9.729
ALT-946	4.723	-0.053	2.335	4.776	0.571	2.608	0.273	2.881
Aminoguanidine	4.929	-0.547	2.191	5.476	0.438	2.315	0.123	2.438
Carnosine	5.569	0.597	3.083	4.971	0.956	3.764	0.681	4.445
GLY-230	4.951	0.997	2.974	3.954	1.119	3.971	0.997	4.968
LR-9	5.216	1.913	3.564	3.303	1.923	5.835	2.271	8.105
Metformin	5.331	-0.181	2.575	5.511	0.602	2.835	0.260	3.095
OPB-9195	5.565	2.055	3.810	3.510	2.068	6.260	2.450	8.710
Pentoxifylline	5.683	1.327	3.505	4.356	1.410	4.846	1.340	6.186
Pioglitazone	5.573	1.447	3.510	4.125	1.493	4.999	1.489	6.489
Pyridoxamine	5.765	0.994	3.379	4.771	1.197	4.382	1.002	5.384
Tenilsetam	5.652	0.681	3.167	4.971	1.008	3.911	0.744	4.655

Table S5B: Descriptors J_I, J_A, J_{HL}, J_χ, J_η, J_ω, J_{D1}, J_{ω^+}, J_{ω^-}, $J_{\Delta\omega^\pm}$ and J_{D2} for the ALT-711, ALT-946, Aminoguanidine, Carnosine, GLY-230, LR-9, Metformin, OPB-9195, Pentoxifylline, Pioglitazone, Pyridoxamine and Tenilsetam molecules calculated from the results of Table S5A

	J_I	J_A	J_{HL}	J_χ	J_η	J_ω	J_{D1}	J_{ω^-}	J_{ω^+}	$J_{\Delta\omega^\pm}$	J_{D2}
ALT-711	0.964	0.385	1.037	0.289	1.348	0.853	1.621	1.476	1.766	3.242	3.975
ALT-946	0.584	0.365	0.688	0.110	0.948	0.076	0.958	0.038	0.148	0.186	0.241
Aminoguanidine	0.648	0.016	0.648	0.332	0.632	0.082	0.718	0.368	0.037	0.405	0.549
Carnosine	0.374	0.471	0.602	0.048	0.845	0.232	0.878	0.436	0.388	0.823	1.009
GLY-230	0.312	0.226	0.385	0.043	0.538	0.139	0.557	0.223	0.266	0.490	0.600
LR-9	0.286	0.286	0.404	0.000	0.571	0.402	0.699	0.769	0.769	1.537	1.883
Metformin	0.523	0.401	0.659	0.061	0.924	0.087	0.930	0.087	0.147	0.234	0.290
OPB-9195	0.279	0.435	0.517	0.078	0.714	0.635	0.958	1.265	1.186	2.451	3.002
Pentoxifylline	0.381	0.348	0.516	0.016	0.730	0.268	0.777	0.482	0.499	0.981	1.201
Pioglitazone	0.380	0.436	0.579	0.028	0.816	0.398	0.909	0.760	0.732	1.492	1.827
Pyridoxamine	0.362	0.424	0.558	0.031	0.786	0.262	0.829	0.491	0.460	0.952	1.166
Tenilsetam	0.421	0.462	0.625	0.020	0.884	0.234	0.914	0.423	0.402	0.825	1.011
Average	0.459	0.355	0.601	0.088	0.811	0.306	0.896	0.568	0.567	1.135	1.396

Table S6A: HOMO and LUMO orbital energies (in eV), ionization potentials I and electron affinities A (in eV), and global electronegativity χ, total hardness η, global electrophilicity ω, electrodonating power (ω^-), electroaccepting power (ω^+), and net electrophilicity $\Delta\omega^\pm$ of ALT-711, ALT-946, Aminoguanidine, Carnosine, GLY-230, LR-9, Metformin, OPB-9195, Pentoxifylline, Pioglitazone, Pyridoxamine and Tenilsetam calculated with the N12SX density functional and the Def2TZVP basis set using water as solvent simulated with the SMD parametrization of the IEF-PCM model. The upper part of the table shows the results derived assuming the validity of the KID procedure and the lower part shows the results derived from the calculated ΔSCF energies.

Property	HOMO	LUMO	χ_K	η_K	ω_K	ω_K^-	ω_K^+	$\Delta\omega_K^\pm$
ALT-711	-5.840	-2.461	4.150	3.379	2.549	7.384	3.233	10.617
ALT-946	-5.030	0.247	2.392	5.277	0.542	2.610	0.218	2.828
Aminoguanidine	-5.112	1.009	2.051	6.122	0.344	2.096	0.044	2.140
Carnosine	-5.943	-0.527	3.235	5.416	0.966	3.888	0.653	4.542
GLY-230	-5.292	-0.892	3.092	4.400	1.086	3.994	0.902	4.895
LR-9	-5.641	-1.898	3.769	3.743	1.898	5.915	2.145	8.060
Metformin	-5.730	0.267	2.732	5.997	0.622	2.985	0.253	3.238
OPB-9195	-6.071	-2.100	4.086	3.971	2.102	6.495	2.409	8.904
Pentoxifylline	-6.033	-1.286	3.660	4.747	1.411	4.948	1.288	6.236
Pioglitazone	-5.945	-1.365	3.655	4.581	1.458	5.031	1.375	6.406
Pyridoxamine	-6.037	-0.935	3.486	5.102	1.191	4.443	0.957	5.401
Tenilsetam	-6.148	-0.770	3.459	5.378	1.112	4.290	0.831	5.122
Property	I	A	χ	η	ω	ω^-	ω^+	$\Delta\omega^\pm$
ALT-711	6.235	2.494	4.364	3.741	2.546	7.508	3.144	10.651
ALT-946	5.048	-0.133	2.458	5.180	0.583	2.718	0.261	2.979
Aminoguanidine	5.103	-0.498	2.302	5.601	0.473	2.448	0.145	2.593
Carnosine	5.900	0.619	3.260	5.281	1.006	3.972	0.712	4.685
GLY-230	5.226	0.966	3.096	4.260	1.125	4.065	0.969	5.034
LR-9	5.617	1.968	3.793	3.650	1.971	6.066	2.273	8.339
Metformin	5.624	-0.193	2.715	5.817	0.634	2.989	0.273	3.262
OPB-9195	6.019	2.155	4.087	3.865	2.161	6.607	2.520	9.127
Pentoxifylline	6.025	1.346	3.685	4.680	1.451	5.038	1.352	6.390
Pioglitazone	5.898	1.408	3.653	4.490	1.486	5.079	1.426	6.505
Pyridoxamine	5.981	1.004	3.493	4.977	1.225	4.508	1.016	5.524
Tenilsetam	6.129	0.802	3.466	5.327	1.127	4.321	0.855	5.176

Table S6B: Descriptors J_I, J_A, J_{HL}, J_χ, J_η, J_ω, J_{D1}, J_{ω^+}, J_{ω^-}, $J_{\Delta\omega^\pm}$ and J_{D2} for the ALT-711, ALT-946, Aminoguanidine, Carnosine, GLY-230, LR-9, Metformin, OPB-9195, Pentoxifylline, Pioglitazone, Pyridoxamine and Tenilsetam molecules calculated from the results of Table S6A

	J_I	J_A	J_{HL}	J_χ	J_η	J_ω	J_{D1}	J_{ω^-}	J_{ω^+}	$J_{\Delta\omega^\pm}$	J_{D2}
ALT-711	0.395	0.033	0.396	0.214	0.361	0.003	0.420	0.124	0.090	0.034	0.157
ALT-946	0.018	0.114	0.115	0.066	0.096	0.041	0.123	0.109	0.043	0.151	0.191
Aminoguanidine	0.010	0.511	0.511	0.251	0.521	0.129	0.592	0.352	0.101	0.453	0.582
Carnosine	0.043	0.092	0.102	0.025	0.135	0.040	0.143	0.084	0.059	0.143	0.176
GLY-230	0.066	0.075	0.100	0.004	0.141	0.039	0.146	0.071	0.067	0.138	0.170
LR-9	0.024	0.070	0.074	0.023	0.093	0.073	0.121	0.151	0.128	0.279	0.342
Metformin	0.107	0.074	0.130	0.017	0.180	0.012	0.181	0.004	0.020	0.024	0.031
OPB-9195	0.052	0.054	0.075	0.001	0.106	0.059	0.122	0.112	0.111	0.224	0.274
Pentoxifylline	0.008	0.059	0.060	0.026	0.067	0.040	0.082	0.090	0.064	0.153	0.189
Pioglitazone	0.047	0.043	0.064	0.002	0.090	0.028	0.095	0.048	0.051	0.099	0.121
Pyridoxamine	0.056	0.069	0.089	0.007	0.125	0.035	0.130	0.065	0.058	0.123	0.151
Tenilsetam	0.019	0.032	0.037	0.007	0.051	0.015	0.053	0.030	0.024	0.054	0.066
Average	0.070	0.102	0.146	0.054	0.164	0.043	0.184	0.103	0.068	0.156	0.204

Table S7A: HOMO and LUMO orbital energies (in eV), ionization potentials I and electron affinities A (in eV), and global electronegativity χ, total hardness η, global electrophilicity ω, electrodonating power (ω^-), electroaccepting power (ω^+), and net electrophilicity $\Delta\omega^\pm$ of ALT-711, ALT-946, Aminoguanidine, Carnosine, GLY-230, LR-9, Metformin, OPB-9195, Pentoxifylline, Pioglitazone, Pyridoxamine and Tenilsetam calculated with the SOGGA11 density functional and the Def2TZVP basis set using water as solvent simulated with the SMD parametrization of the IEF-PCM model. The upper part of the table shows the results derived assuming the validity of the KID procedure and the lower part shows the results derived from the calculated ΔSCF energies.

Property	HOMO	LUMO	χ_K	η_K	ω_K	ω_K^-	ω_K^+	$\Delta\omega_K^\pm$
ALT-711	-5.129	-2.934	4.032	2.195	3.702	9.557	5.526	15.083
ALT-946	-4.267	-0.561	2.414	3.706	0.786	3.011	0.597	3.608
Aminoguanidine	-4.302	0.225	2.039	4.527	0.459	2.220	0.182	2.402
Carnosine	-5.501	-1.571	3.536	3.930	1.590	5.195	1.659	6.853
GLY-230	-4.899	-1.565	3.232	3.335	1.567	4.958	1.725	6.683
LR-9	-5.154	-2.398	3.776	2.756	2.587	7.235	3.459	10.694
Metformin	-5.029	-0.991	3.010	4.038	1.122	4.000	0.991	4.991
OPB-9195	-5.422	-2.649	4.036	2.774	2.936	8.063	4.027	12.090
Pentoxifylline	-5.451	-1.925	3.688	3.526	1.928	5.921	2.233	8.154
Pioglitazone	-5.413	-2.209	3.811	3.204	2.266	6.638	2.827	9.465
Pyridoxamine	-5.535	-1.714	3.624	3.821	1.719	5.489	1.864	7.353
Tenilsetam	-5.331	-1.466	3.398	3.865	1.494	4.929	1.531	6.460
Property	I	A	χ	η	ω	ω^-	ω^+	$\Delta\omega^\pm$
ALT-711	6.102	2.528	4.315	3.574	2.605	7.591	3.276	10.867
ALT-946	4.894	0.366	2.630	4.528	0.764	3.126	0.496	3.621
Aminoguanidine	5.040	-0.345	2.347	5.386	0.512	2.533	0.186	2.719
Carnosine	5.828	1.104	3.466	4.724	1.271	4.571	1.105	5.676
GLY-230	5.231	1.320	3.276	3.910	1.372	4.626	1.350	5.977
LR-9	5.481	2.115	3.798	3.366	2.143	6.395	2.597	8.992
Metformin	5.591	-0.104	2.743	5.694	0.661	3.049	0.306	3.355
OPB-9195	5.740	2.191	3.965	3.549	2.215	6.635	2.670	9.305
Pentoxifylline	5.836	1.595	3.716	4.241	1.628	5.378	1.663	7.041
Pioglitazone	5.815	1.724	3.769	4.091	1.736	5.613	1.844	7.457
Pyridoxamine	5.930	1.375	3.652	4.554	1.465	5.040	1.388	6.428
Tenilsetam	5.819	0.513	3.166	5.306	0.945	3.804	0.638	4.442

Table S7B: Descriptors J_I, J_A, J_{HL}, J_χ, J_η, J_ω, J_{D1}, J_{ω^+}, J_{ω^-}, $J_{\Delta\omega^\pm}$ and J_{D2} for the ALT-711, ALT-946, Aminoguanidine, Carnosine, GLY-230, LR-9, Metformin, OPB-9195, Pentoxifylline, Pioglitazone, Pyridoxamine and Tenilsetam molecules calculated from the results of Table S7A

	J_I	J_A	J_{HL}	J_χ	J_η	J_ω	J_{D1}	J_{ω^-}	J_{ω^+}	$J_{\Delta\omega^\pm}$	J_{D2}
ALT-711	0.973	0.406	1.054	0.284	1.379	1.097	1.785	1.966	2.250	4.216	5.167
ALT-946	0.626	0.195	0.656	0.216	0.821	0.022	0.850	0.114	0.101	0.013	0.153
Aminoguanidine	0.738	0.120	0.747	0.309	0.858	0.053	0.913	0.313	0.004	0.317	0.446
Carnosine	0.327	0.467	0.570	0.070	0.794	0.319	0.859	0.623	0.554	1.177	1.442
GLY-230	0.331	0.245	0.412	0.043	0.576	0.195	0.609	0.331	0.375	0.706	0.866
LR-9	0.326	0.283	0.432	0.021	0.610	0.445	0.755	0.840	0.862	1.702	2.085
Metformin	0.562	1.094	1.230	0.266	1.656	0.461	1.739	0.951	0.685	1.636	2.012
OPB-9195	0.317	0.458	0.557	0.070	0.775	0.720	1.061	1.427	1.357	2.785	3.411
Pentoxifylline	0.385	0.329	0.507	0.028	0.715	0.301	0.776	0.543	0.571	1.113	1.364
Pioglitazone	0.402	0.485	0.630	0.042	0.887	0.530	1.034	1.025	0.983	2.009	2.460
Pyridoxamine	0.395	0.339	0.520	0.028	0.734	0.254	0.777	0.449	0.477	0.925	1.134
Tenilsetam	0.489	0.953	1.071	0.232	1.442	0.550	1.560	1.125	0.893	2.018	2.477
Average	0.489	0.448	0.699	0.134	0.937	0.412	1.060	0.809	0.759	1.551	1.918

Table S8A: HOMO and LUMO orbital energies (in eV), ionization potentials I and electron affinities A (in eV), and global electronegativity χ, total hardness η, global electrophilicity ω, electrodonating power (ω^-), electroaccepting power (ω^+), and net electrophilicity $\Delta\omega^\pm$ of ALT-711, ALT-946, Aminoguanidine, Carnosine, GLY-230, LR-9, Metformin, OPB-9195, Pentoxifylline, Pioglitazone, Pyridoxamine and Tenilsetam calculated with the SOGGA11X density functional and the Def2TZVP basis set using water as solvent simulated with the SMD parametrization of the IEF-PCM model. The upper part of the table shows the results derived assuming the validity of the KID procedure and the lower part shows the results derived from the calculated ΔSCF energies.

Property	HOMO	LUMO	χ_K	η_K	ω_K	ω_K^-	ω_K^+	$\Delta\omega_K^\pm$
ALT-711	-7.122	-1.539	4.330	5.583	1.679	5.872	1.542	7.415
ALT-946	-6.807	1.214	2.797	8.021	0.488	2.875	0.078	2.953
Aminoguanidine	-6.606	1.845	2.380	8.451	0.335	2.389	0.008	2.397
Carnosine	-7.010	0.540	3.235	7.550	0.693	3.475	0.240	3.716
GLY-230	-6.292	-0.106	3.199	6.186	0.827	3.640	0.441	4.082
LR-9	-6.674	-1.099	3.886	5.575	1.355	5.001	1.114	6.115
Metformin	-7.075	1.025	3.025	8.100	0.565	3.149	0.124	3.272
OPB-9195	-7.120	-1.461	4.290	5.659	1.626	5.752	1.461	7.213
Pentoxifylline	-7.058	-0.354	3.706	6.704	1.024	4.321	0.615	4.936
Pioglitazone	-7.025	-0.337	3.681	6.688	1.013	4.285	0.604	4.888
Pyridoxamine	-7.098	-0.118	3.608	6.980	0.933	4.105	0.497	4.603
Tenilsetam	-7.306	0.063	3.621	7.370	0.890	4.051	0.429	4.480
Property	I	A	χ	η	ω	ω^-	ω^+	$\Delta\omega^\pm$
ALT-711	6.413	2.362	4.387	4.052	2.375	7.198	2.810	10.008
ALT-946	5.767	-0.162	2.803	5.929	0.662	3.097	0.294	3.391
Aminoguanidine	5.534	-0.630	2.452	6.164	0.488	2.586	0.135	2.721
Carnosine	6.033	0.581	3.307	5.452	1.003	4.001	0.693	4.694
GLY-230	5.410	0.937	3.174	4.474	1.126	4.118	0.944	5.062
LR-9	5.819	1.941	3.880	3.877	1.941	6.065	2.185	8.250
Metformin	5.930	-0.071	2.929	6.001	0.715	3.270	0.340	3.610
OPB-9195	6.217	2.357	4.287	3.860	2.381	7.147	2.860	10.007
Pentoxifylline	6.164	1.285	3.724	4.879	1.421	5.010	1.286	6.295
Pioglitazone	6.085	1.291	3.688	4.795	1.419	4.981	1.293	6.273
Pyridoxamine	6.144	1.065	3.604	5.078	1.279	4.678	1.074	5.752
Tenilsetam	6.353	0.865	3.609	5.487	1.187	4.521	0.912	5.433

Table S8B: Descriptors J_I, J_A, J_{HL}, J_χ, J_η, J_ω, J_{D1}, J_{ω^+}, J_{ω^-}, $J_{\Delta\omega^\pm}$ and J_{D2} for the ALT-711, ALT-946, Aminoguanidine, Carnosine, GLY-230, LR-9, Metformin, OPB-9195, Pentoxifylline, Pioglitazone, Pyridoxamine and Tenilsetam molecules calculated from the results of Table S8A

	J_I	J_A	J_{HL}	J_χ	J_η	J_ω	J_{D1}	J_{ω^-}	J_{ω^+}	$J_{\Delta\omega^\pm}$	J_{D2}
ALT-711	0.709	0.823	1.086	0.057	1.532	0.696	1.683	1.325	1.268	2.594	3.177
ALT-946	1.040	1.052	1.479	0.006	2.092	0.175	2.099	0.222	0.216	0.438	0.537
Aminoguanidine	1.072	1.215	1.620	0.071	2.287	0.152	2.293	0.197	0.126	0.324	0.399
Carnosine	0.977	1.122	1.488	0.073	2.099	0.310	2.123	0.526	0.453	0.979	1.200
GLY-230	0.881	0.831	1.211	0.025	1.712	0.299	1.738	0.477	0.503	0.980	1.200
LR-9	0.855	0.843	1.201	0.006	1.698	0.587	1.796	1.064	1.071	2.135	2.615
Metformin	1.145	0.953	1.490	0.096	2.098	0.150	2.106	0.121	0.217	0.338	0.419
OPB-9195	0.903	0.897	1.272	0.003	1.799	0.755	1.951	1.396	1.399	2.794	3.422
Pentoxifylline	0.895	0.931	1.291	0.018	1.825	0.397	1.868	0.689	0.671	1.360	1.666
Pioglitazone	0.940	0.954	1.339	0.007	1.893	0.405	1.936	0.696	0.689	1.385	1.697
Pyridoxamine	0.955	0.947	1.345	0.004	1.902	0.347	1.933	0.573	0.576	1.149	1.407
Tenilsetam	0.954	0.929	1.331	0.012	1.883	0.297	1.906	0.470	0.483	0.953	1.167
Average	0.944	0.958	1.346	0.032	1.902	0.381	1.953	0.646	0.639	1.286	1.575
